# Checking procalcitonin suitability for prognosis and antimicrobial therapy monitoring in burn patients

**DOI:** 10.1186/s41038-018-0112-5

**Published:** 2018-03-31

**Authors:** Luís Cabral, Vera Afreixo, Rita Meireles, Miguel Vaz, Catarina Chaves, Marisa Caetano, Luís Almeida, José Artur Paiva

**Affiliations:** 1 Department of Plastic Surgery and Burns Unit, Unidade de Queimados, Coimbra University Hospital Centre (CHUC), Av. Bissaya Barreto s/n, 3000-075 Coimbra, Portugal; 2 Autonomous Section of Health Sciences (SACS), University of Aveiro, Aveiro, Portugal; 3 CIDMA - Center for Research and Development in Mathematics and Applications, iBiMED, Institute for Biomedicine, University of Aveiro, Aveiro, Portugal; 4 Clinical Pathology Department, Coimbra University Hospital Centre (CHUC), Coimbra, Portugal; 5 Pharmacy Department, Coimbra University Hospital Centre (CHUC), Coimbra, Portugal; 6 MedinUP, Department of Pharmacology and Therapeutics, Faculty of Medicine, University of Porto, Porto, Portugal; 7 Department of Emergency and Intensive Care Medicine, Centro Hospitalar São João, Porto, Portugal; 8 Faculty of Medicine, University of Porto, Grupo de Infecção e Sépsis, Porto, Portugal

**Keywords:** Burns, Sepsis, Procalcitonin, Prognosis, Antimicrobial stewardship

## Abstract

**Background:**

Due to greater infection susceptibility, sepsis is the main cause of death in burn patients. Quick diagnosis and patient stratification, early and appropriated antimicrobial therapy, and focus control are crucial for patients’ survival. On the other hand, superfluous extension of therapy is associated with adverse events and arousal of microbial resistance. The use of biomarkers, necessarily coupled with close clinical examination, may predict outcomes, stratifying patients who need more intensive care, and monitor the efficacy of antimicrobial therapy, allowing faster de-escalation or stop, reducing the development of resistance and possibly the financial burden, without increasing mortality. The aim of this work is to check the suitability of procalcitonin (PCT) to fulfill these goals in a large sample of septic burn patients.

**Methods:**

One hundred and one patients, with 15% or more of total body surface area (TBSA) burned, admitted from January 2011 to December 2014 at Coimbra Burns Unit (CBU), in Portugal were included in the sample. All patients had a diagnosis of sepsis, according to the American Burn Association (ABA) criteria. The sample was factored by survival (68 survivors and 33 non-survivors). The maximum value of PCT in each day was used for statistical analysis. Data were summarized by location measures (mean, median, minimum, maximum, quartiles) and dispersion measures (standard error and range measures). Statistical analysis was performed with SPSS© 23.0 IBM© for Windows©.

**Results:**

There were statistically significant differences between PCT levels of patients from the survivor and non-survivor groups during the first and the last weeks of hospitalization as well as during the first week after sepsis suspicion, being slightly higher during this period. During the first 7 days of antimicrobial therapy, PCT was always higher in the non-survivor, still without reaching statistical significance, but when the analysis was extended till the 15th day, PCT increased significantly, rapidly, and steadily, denouncing therapy failure.

**Conclusion:**

Despite being not an ideal biomarker, PCT proved to have good prognostic power in septic burn patients, paralleling the evolution of the infectious process and reflecting the efficacy of antimicrobial therapy, and the inclusion of its serial dosing may be advised to reinforce antimicrobial stewardship programs at burn units; meanwhile, more accurate approaches are not available.

**Electronic supplementary material:**

The online version of this article (10.1186/s41038-018-0112-5) contains supplementary material, which is available to authorized users.

## Background

Sepsis is still nowadays the main cause of death in burn patients due to the impact of extensive burns in all organ systems, affecting homeostatic mechanisms, and to the greater susceptibility of this population to infection [1, 2], related to the loss of the cutaneous barrier, immunosuppression, use of invasive devices, nosocomial flora, etc. Survival is directly dependent on the institution of prompt and adequate antimicrobial therapy [3]. However, the gold standard for sepsis diagnosis still relies on the identification of microorganisms in blood cultures, which unfortunately are positive only in 20–30% of all confirmed bloodstream infections, and their results may take 48 to 72 h to reach the prescriber [4]. While more rapid methods of microbiological identification, such as polymerase-chain reaction (PCR) [5], matrix-assisted laser desorption ionization–time of flight mass spectrometry (MALDI-TOF MS), gene expression profiling, aptamer panels, etc. [6], are not either widely available or fully developed, the use of early empirical often broad-spectrum antibiotic therapy is warranted. This empirical strategy increases the likelihood of cure of infection and survival but negatively impacts in terms of microbiome, leading to the selection and emergence of antimicrobial resistance. In this context, biochemical biomarkers, namely procalcitonin (PCT) alone [7, 8] or integrating a composite panel [9–12], and always coupled with thorough clinical examination, may be an important aid for the early suspicion of sepsis and rapid institution of therapy, which is strongly associated with improved outcomes [13, 14].

PCT is a 116-amino acid precursor of calcitonin, which synthesis and secretion, encoded by first calcitonin gene (CALC-I gene), and normally restricted to thyroid C cells and some neuroendrocrine cells of the lungs and gut, is upregulated by the presence in the blood of microbial toxins, necrotic body cells, and some proinflammatory cytokines (IL-1, IL-6, TNF-α, etc.), in a synergistic way, starting to be produced in great amounts by many other nonendocrine types of cells, including monocytes and adipocytes [15], reaching measurable levels in 2–4 h after onset of the infectious process, peaking at 24–30 h, and rapidly subsiding with recovery. PCT increment is less pronounced with fungal infection and is absent in viral disease, allegedly due to inhibition of its secretion by some cytokines released as a response to viral infection, like interferon-γ [16].

Besides its utility to help clinicians in the diagnosis of sepsis [17] including patients admitted to burn units [18], the magnitude and duration of PCT elevation seems to correlate with injury severity and outcome, and there are several published works analyzing its potential for the prognosis and for the monitoring of antimicrobial therapy, helping decisions on early antibiotic de-escalation or rescue therapy [19–21]. Most of these are focused in lower respiratory tract infections and/or intensive care patients, while papers on septic burn patients are scarce [22].

The purpose of this work is to evaluate the feasibility of PCT use to predict the outcome and to monitor the efficacy of antimicrobial therapy in a sample of severe adult burn patients.

## Methods

The sample under analysis was composed by 101 burn patients, with 15% or more of total body surface area (TBSA) burned, admitted from January 2011 to December 2014 at Coimbra Burns Unit (CBU), a department of Coimbra Hospital and University Center (CHUC), in Portugal. Being a retrospective observational study of patients from a suitably anonymized dataset, involving only recording data from the medical record, the Ethics Committee from CHUC, according to the Declaration of Helsinki and Council for International Organizations of Medical Sciences (CIOMS) International Ethics Guidelines, waived the need of informed consent.

All the patients had a diagnosis of sepsis. This diagnosis was done according to the American Burn Association (ABA) criteria [23]: a clinical suspicion of infection coupled with the presence of three or more of the following parameters: temperature > 39 or < 36.5 °C; tachycardia > 110 beats per minute; tachypnea > 25 breaths per minute or minute ventilation > 12 L/min; thrombocytopenia < 100,000/μL; hyperglycemia (untreated plasma glucose > 200 mg/dL or intravenous glucose requirement > 7 U/h over 24 h); and enteral feeding intolerance: abdominal distension or gastric residuals more than two times feeding rate or diarrhea > 2500 mL/24 h.

PCT was measured with time-resolved amplified cryptate emission (TRACE) technology (Kryptor PCT; Brahms AG; Hennigsdorf, Germany). The sample was factored by survival (68 survivors and 33 non-survivors). The maximum value of PCT in each day of the study was used for statistical analysis and when samples were not collected in some days (till a maximum of 5 days), the missing values of the interval were calculated as the median value between the PCT determinations available.

### Statistical analysis

Data were summarized by location measures (mean, median, minimum, maximum, quartiles) and dispersion measures (standard error and range measures).

The variables under study present a non-Gaussian distribution. Under a nonparametric approach, the quantitative variables were compared with the Mann-Whitney U tests and qualitative variables were compared with the Pearson chi-square test. Time variations of PCT levels were tested using Friedman’s test and Kendall’s *W* ranges from 0 (no agreement) to 1 (complete agreement).

To measure the difference effect size between the two independent groups, the probability of superiority (PS) was used. PS ranges from 0.0 to 1.0 and PS = 0.5 state that there are no differences between the groups [A] and PS = 0 or PS = 1 states the maximum effect.

Statistical analysis was performed with SPSS© 23.0 IBM© for Windows©, and in a statistical hypothesis test, a *p* value ≤ 0.05 means the effect was considered significant.

## Results

### Sample description

Population characteristics are described in Table 1. After factorization by survival, a significant heterogeneity was found between the two groups (68 survivors and 33 non-survivors) for the age of the patients, the Abbreviated Burn Severity Index (ABSI) score (Additional file 1) [24], the TBSA burned, the presence of inhalation injury, the need of mechanical ventilation and its duration, the number of surgical interventions, the duration of sepsis episode, and the length of the stay at the burn unit. Heterogeneity was not found for gender, burn degree, and duration of antimicrobial therapy.

**Table 1:** Patients’ characteristics

Characteristics	Survivors	Non-survivors	*p* value
Number of patients	68	33	
Age (years)	53.0 ± 2.4 (18–85)	70.5 ± 3.5 (28–90)	0.000*
Male, gender (%)	38 (55.9%)	19 (57.6%)	0.872
Burn degree (2nd/2nd and 3rd/3rd)	8/50/10	1/24/8	0.219
ABSI score	8.0 ± 0.2 (4–13)	10.4 ± 0.4 (8–17)	0.000*
TBSA burned (%)	28.2 ± 1.6 (14–75)	40.7 ± 3.6 (15–90)	0.000*
Inhalation injury (%)	45 (66.2%)	15 (45.5%)	0.047*
Mechanical ventilation (%)	36 (52.9%)	4 (12.1%)	0.000*
Days of mechanical ventilation	11.3 ± 2.3 (0–70)	22.4 ± 3.8 (0–76)	0.000*
Duration of sepsis episode (days)	5.5 ± 0.6 (1–24)	10.6 ± 1.8 (1–43)	0.005*
Antimicrobial therapy (days)	20.8 ± 2.4 (0–104)	18.5 ± 3.4 (0–64)	0.374
Number of surgical interventions	4.3 ± 0.3 (0–15)	2.5 ± 0.6 (0–12)	0.000*
Length of stay (days)	43.1 ± 3.2 (8–180)	29.9 ± 5.0 (3–113)	0.001*

Values are mean ± S.E. (min-max)

*Significant difference at *p* value < 0.05

*ABSI* Abbreviated Burn Severity Index, *TBSA* Total body surface area, *S.E*. Standard error

Table 2 shows the comparison of individual PCT location measures, presenting significant differences between survivors and non-survivors in all statistical parameters (minimum, median, mean, maximum).

**Table 2:** Analysis of individual procalcitonin (PCT) location measures in survivor and non-survivor patients, showing statistically significant differences for all parameters

	Survivors	Non-survivors	*p* value
PCT minimum	0.10 ± 0.01 (0.02–0.39)	2.84 ± 1.59 (0.06–48.39)	0.000*
PCT median	0.57 ± 0.10 (0.05–4.31)	4.73 ± 1.93 (0.27–58.99)	0.000*
PCT mean	2.04 ± 0.48 (0.05–26.28)	7.00 ± 1.98 (0.05–58.99)	0.000*
PCT maximum	18.40 ± 4.38 (0.07–237.60)	28.07 ± 5.98 (0.87–145.40)	0.002*

Values are mean ± S.E. (min-max)

*Significant difference at *p* value < 0.05

*PCT* procalcitonin, *S.E*. Standard error

**Fig. 1:**
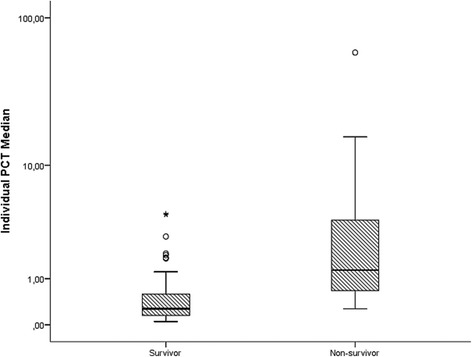
Box plots of individual procalcitonin (PCT) median according to survivor and non-survivor groups. *p < 0.05 means significant differences

The box plots of individual median PCT levels for each group are presented in Fig. 1, being significantly lower for survivors.

### PCT evolution along the first week of stay

**Fig. 2:**
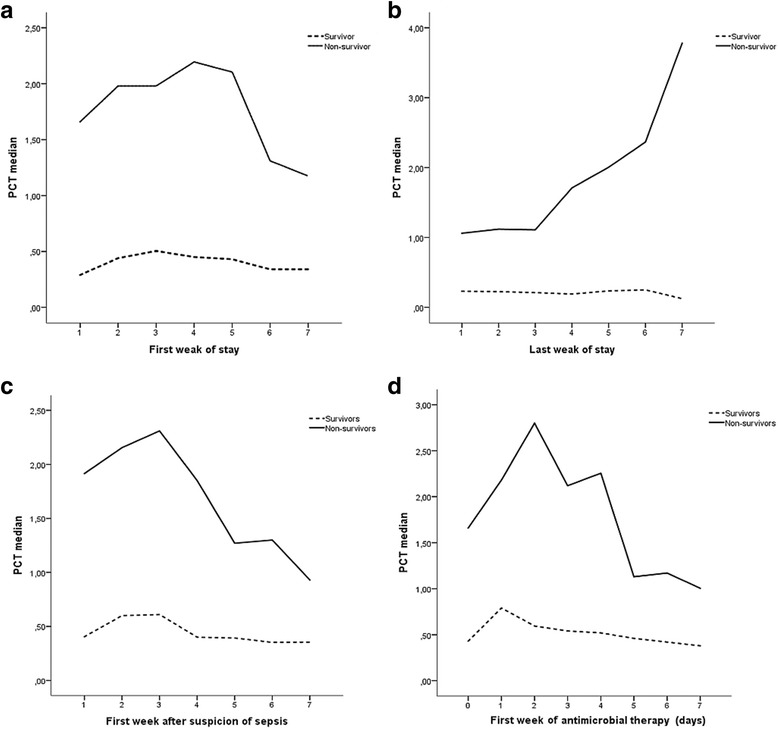
Line plots of procalcitonin (PCT) levels evolution along the first week of hospitalization (**a**), last week of hospitalization (**b**), first week after suspicion of sepsis (**c**), and first week of antimicrobial therapy (**d**), showing significant differences between survivor and non-survivor groups in **a**, **b**, **c**, **d**

Table 3 shows the evolution PCT levels in patients from the survivor and non-survivor groups during the first week of stay at CBU. The data presents missing values of the PCT in some of the days of hospitalization and this is the reason for this variation in the number of individuals by scenario. Differences between PCT levels of patients from the survivor and non-survivor groups during the first week of hospitalization are statistically significant (Fig. 2a and Table 6).

**Table 3:** Evolution of procalcitonin levels during the first week of hospitalization for survivor and non-survivor groups

First week of hospitalization						
	Survivors			Non-survivors		
Day	*N*	Median	Q1–Q3	*N*	Median	Q1–Q3
1	58	0.290	0.150–1.160	27	1.6600	0.405–7.995
2	65	0.345	0.170–1.650	28	2.0550	0.270–6.840
3	66	0.360	0.170–1.640	27	1.9800	0.565–3.220
4	67	0.420	0.175–1.155	26	2.0550	0.520–4.170
5	67	0.345	0.160–0.830	26	1.7900	0.700–4.370
6	68	0.330	0.155–0.785	25	1.3100	0.560–2.850
7	68	0.360	0.160–0.985	24	1.7000	0.730–5.555

*Q1-Q3* 1^st^ Quartile- 3^rd^ Quartile

### PCT evolution along the last week of stay

The evolution of PCT levels for survivor and non-survivor groups in their last week of stay at CBU is presented in Table 4. A statistically significant difference was also demonstrated for this period of time (Fig. 2b and Table 6).

**Table 4:** Evolution of procalcitonin levels in the last week of hospitalization for survivor and non-survivor groups

Last week of hospitalization						
	Survivors			Non-survivors		
Day	*N*	Median	Q1–Q3	*N*	Median	Q1–Q3
1	67	0.180	0.100–0.395	22	1.050	0.700–2.370
2	68	0.160	0.095–0.435	24	1.0150	0.435–2.830
3	68	0.150	0.080–0.400	26	1.1100	0.560–2.510
4	68	0.160	0.080–0.320	26	1.2000	0.460–2.825
5	68	0.150	0.070–0.355	27	1.4700	0.650–3.570
6	68	0.140	0.070–0.360	28	2.3650	0.710–5.820
7	68	0.125	0.070–0.365	31	3.8200	1.100–10.235

*Q1-Q3* 1^st^ Quartile- 3^rd^ Quartile

### PCT evolution in the first week after suspicion of sepsis

A statistical analysis of PCT evolution in the first week after suspicion of sepsis, as defined by ABA criteria, was also carried out. Data are presented in Table 5. A significant difference between survivor and non-survivor groups was detected (Fig. 2c and Table 6).

**Table 5:** Evolution of procalcitonin levels during the first week after suspicion of sepsis for survivor and non-survivor groups

First week after suspicion of sepsis						
	Survivors			Non-survivors		
Day	*N*	Median	Q1–Q3	*N*	Median	Q1–Q3
1	58	0.385	0.160–2.260	26	1.915	0.460–6.170
2	63	0.600	0.200–2.430	27	2.100	0.560–6.735
3	65	0.610	0.200–2.120	26	2.310	0.550–5.610
4	65	0.400	0.200–1.170	24	1.850	0.485–5.965
5	64	0.395	0.210–1.160	23	1.270	0.545–3.820
6	60	0.345	0.170–1.240	21	1.300	0.720–4.910
7	52	0.330	0.160–1.470	20	0.930	0.515–2.350

*Q1-Q3* 1^st^ Quartile- 3^rd^ Quartile

**Table 6:** Comparison between survivors and non-survivors during three periods of stay (first week of stay, last week of stay, and first week after suspicion of sepsis)

Period	Survivors			Non-Survivors			Global difference
	*p* value^a^	Kendall’s *W*	*N*	*p* value^a^	Kendall’s *W*	*N*	*p* value^b^
First week of stay	0.925	0.006	58	0.504	0.042	21	0.000*
Last week of stay	0.000*	0.162	67	0.050	0.095	22	0.000*
First week after suspicion of sepsis	0.000*	0.117	46	0.217	0.077	18	0.002*

Significant difference (*p value < 0.05)

^a^Friedman test *p* value

^b^The minimum *p* value of all simultaneous Mann-Whitney U tests with Sidak correction

In order to compare the relative prognostic value of PCT levels in each of the abovementioned periods (first week of hospitalization, last week of hospitalization, and first week after sepsis suspicion), statistical tests were done, namely Friedman test *p* value and Mann-Whitney U test *p* values with Sidak correction (Table 6).

Furthermore, the PS effect [25] was determined. The results are transcribed in Table 7.

**Table 7:** Probability of superiority (PS) effect in procalcitonin levels due to mortality in different periods of stay (first week of stay, last week of stay, and first week after suspicion of sepsis)

PS effect	D1	D2	D3	D4	D5	D6	D7
First week of stay	0.29	0.26	0.25	0.25	0.21	0.24	0.25
Last week of stay	0.06	0.10	0.13	0.15	0.16	0.20	0.15
First weak after suspicion of sepsis	0.32	0.30	0.31	0.28	0.28	0.24	0.29

### PCT evolution with antimicrobial therapy

**Fig. 3:**
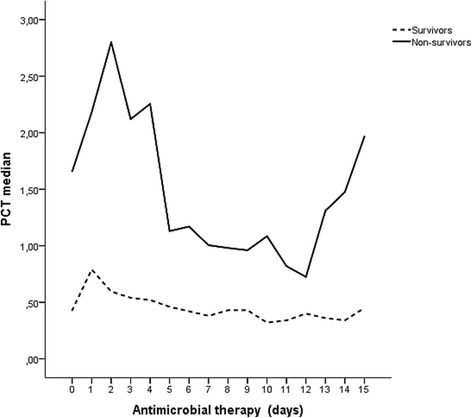
Line plots of procalcitonin (PCT) evolution in the first 15 days of antimicrobial therapy

No statistically significant difference was found between the groups, but a within-group significant variation was detected, with a progressive decline along the first 7 days, supposedly due to antimicrobial action (Fig. 2d and Table 8). When the analysis was extended to the 15th day, it was found that PCT levels increased rapidly and steadily until the day of death in non-survivors, what did not happen in the survivor group, as seen in Fig. 3.

**Table 8:** Evolution of procalcitonin levels in the first week of antimicrobial therapy for survivor and non-survivor groups

First week of antimicrobial therapy						
Day	Survivors			Non-survivors		
	*N*	Median	Q1–Q3	*N*	Median	Q1–Q3
1	66	0.6300	0.240–3.020	24	2.080	0.945–2.810
2	68	0.5500	0.225–2.475	24	2.680	0.870–5.155
3	66	0.4950	0.220–1.300	24	1.945	0.750–7.205
4	66	0.4400	0.220–1.140	23	2.010	0.940–4.485
5	65	0.3500	0.170–1.140	22	1.065	0.550–4.490
6	61	0.3700	0.170–1.250	21	1.070	0.380–3.270
7	59	0.3700	0.175–0.920	21	0.960	0.660–2.420

*Q1-Q3* 1^st^ Quartile- 3^rd^ Quartile

## Discussion

Even acknowledging all advances in critical care, extensive burns are still associated with high morbidity and mortality mainly due to septic episodes [26, 27]. In the last years, diverse studies were published showing the utility of dosing PCT levels as an aid to the diagnosis of systemic infection in burn patients [28–34], particularly when a dynamic approach is used [35]. Notwithstanding the core decision should rely on the clinical features and never on a biomarker alone [36], PCT dosing may support the suspect of ongoing and uncontrolled systemic infection when its values keep rising, or at least does not subside in consecutive analysis, indicating that something must be done to control a probable septic process before it can lead to irreversible damage. Apart its potential to improve clinicians’ diagnostic capacity, PCT has been used with success at the emergency departments [37, 38], to predict the prognosis of suspected septic patients and to stratify them according to the risk of death and the necessity of admission in intensive care units (ICU) [39–41]. PCT levels at admission and, much more reliable [42], its evolution on subsequent days may give insights on the ultimate outcome, which is crucial to clinical management and may be of great importance to inform patient’s relatives and for judicial concerning [43–47]. This valuable predictive power was not found for C-reactive protein (CRP) or white blood cells counting, another currently employed blood biomarkers [48–53]. The prognostic power of PCT dosing has also been stated for burned patients by Kim et al. [54] who, in a prospective observational study with a cohort of 175 patients, showed a significant correlation between PCT levels and mortality rate. In this context, it is worth to note, as referred by Piroglu et al. [55], that clinical scoring systems used to predict mortality of intensive care patients, like Acute Physiology and Chronic Health Evaluation Score II (APACHE II), Simplified Acute Physiology Score (SAPS), Sequential Organ Failure Assessment (SOFA), and Pediatric Risk of Mortality (PRISM), do not include parameters specific for burn patients, and these authors showed that combination of the former score with PCT significantly increased its accuracy. A prospectively study of Lavrentieva et al. [31], including 145 patients, concluded that the maximum PCT level has prognostic value in burn patients, and Mokline et al. [32] found a close correlation of PCT levels with sepsis severity, showing that increasing values were linked with worse outcomes and vice versa.

Another important use of PCT dosing is guiding antimicrobial therapy in septic ICU patients, which is becoming generally accepted [56], supported by several trials [57–62], systematic reviews, and meta-analysis [63–67]; however, some authors still consider that more studies on its safety and efficacy are needed yet [68, 69]. Once a clinical suspicion of sepsis is done, and in particular if corroborated by abnormally elevated PCT levels, empirical antimicrobial therapy, coupled with focus control when feasible, must be immediately started because survival is mostly depending on it and any delay, even hourly, is directly related with an increase in mortality [13, 70, 71]. On the other hand, there is an overwhelming acceptation that a lengthening of antimicrobial therapy beyond that strictly necessary to control the infectious process favors the development of microbial resistance, contributing to the soaring public health risk of having each time less sensitive microorganisms and lack of antimicrobials to combat them [72]. Many published works describe PCT kinetics as a mirror of the evolution of the infectious episode [73–75] as well as a trustable indicator of the antimicrobial therapy efficacy, allowing an early de-escalation and/or stopping of drug administration when its levels progress and consistently subside [76, 77]. When PCT levels keep elevated or even increasing, this is a sound indication that therapy is not working and/or that there are still infectious foci to clean, and if the situation is not rapidly controlled, a bad outcome is foreseeable.

Several authors have discussed in recent works this use of PCT, and a body of evidence is growing to support this approach. Jensen et al. in a trial (PASS Study) [78] published in 2011 advised against PCT-guided antimicrobial escalation, linking it to increased organ-related harm and length of stay at the ICU, without improvement in the outcomes. However, the sample analyzed came from just one developed country with antimicrobial restriction and a traditionally low microbial resistance. On the other hand, focus was not put on the possibility of using PCT levels to help decision on antibiotherapy discontinuation neither a subgroup analysis on burn patients was done. Nevertheless, and even if antimicrobial escalation may be somewhat controversial, PCT has proven to be very useful to monitor antimicrobials efficacy, with its levels paralleling clinical evolution, and to indicate when it is safe to stop it without prejudice to the patients [79]. Indeed, this methodology has proved to safely decrease antimicrobial consumption [80] by reducing days of antimicrobial therapy with strong potential to lower resistance development. This approach has already been validated for use in ICUs, with proven reduction of antimicrobial consumption without increase in morbidity or mortality [81]. Indeed, de Jong et al., in the largest prospective study in ICU patients published to date (SAPS Study) [61], were even able to show a significant reduction of mortality rate. The ever wider diffusion of PCT test, reducing its costs, and its efficacy in this setting, made also possible for some authors to consider it as probably cost-effective [82–86]. In a recent paper, Lavrentieva et al. [87] reported significantly shorter durations of antibiotic treatment in a PCT-guided group of burns patients compared to controls without differences in main outcome characteristics, including mortality rate, length of mechanical ventilation, and length of stay.

Among the limitations of this study are naturally its single-center, retrospective observational character as well as lacking of subgroup analysis according to concomitant pathologies. The definition of a precise *cutoff* of PCT levels for predicting outcomes or stopping antimicrobial therapy was also beyond the scope of this analysis and, as recognized in the literature, it will always be dependent on patient characteristics and facility features, and it is PCT kinetics that deserved authors attention, in spite of 100 ng/mL was often taken as an alert signal. On the positive aspects are the sample size and the strict use of ABA burn sepsis definitions for inclusion criteria. The strength of results from the present study would be largely enhanced by a desirable prospective multicentric trial.

The use of prognostic biomarkers in order to predict outcomes as well for guiding antimicrobial therapy in sepsis patients is nowadays a common practice in intensive care wards. As anytime more acknowledged in the literature, antimicrobial stewardship programs employing current available biomarkers or preferably, a panel of diverse ones, always associated with repeated clinical evaluation, may decisively improve patients’ stratification and antimicrobial use, optimizing patients outcome, reducing the spread of microbial resistance, and cutting financial burden [88–93]. Meanwhile more sophisticated and individualized system-based (integrating genomics, metabolomics, and proteomics) [94–96] data are not available to more accurately predict outcomes and tailor treatment options for burn victims, as well as other intensive care patients, PCT dosing will remain one of the more useful tools to help clinicians decisions.

## Conclusion

In spite of its limitations, the close correlation between PCT levels and patients’ outcomes statistically demonstrated in the present work backs its use for prognosis determination in severe burn patients. Additionally, this study showed that the persistency of abnormally elevated PCT along the days of antimicrobial therapy was linked with poor outcomes in this set of patients, opposed to what happens when their levels fall in a consistent way, reflecting its efficacy.

Prospective multicentric studies would surely give more strength to the generalization of PCT use for prognosis and antimicrobial stewardship in burn patients and are much needed.

## Additional file

Additional file 1: **Annex 1.** Abbreviated Burn Severity Index. (DOCX 19 kb)
